# Recombinant LPG3 Stimulates IFN-Γ and TNF-Α Secretion by Human NK Cells

**Published:** 2015

**Authors:** Sanaz RASOLZADEH, Mostafa HAJI FATAHALIHA, Maryam HOSSEINI, Reza JAFARI, Abolfazl MIAHIPOUR, Sevil SADREDDINI, Zohreh BABALO, Hossein SAMADI KAFIL, Mehdi YOUSEFI

**Affiliations:** 1 *Drug Applied Research Center, Tabriz University of Medical Sciences, Tabriz, Iran*; 2 *Immunology Research Center, Tabriz University of Medical Sciences, Tabriz, Iran*; 3 *Dept. of Immunology, Faculty of Medicine, Tabriz University of Medical Sciences, Tabriz, Iran*; 4 *Dept. of Medical Parasitology and Mycology, School of Medicine, Alborz University of Medical Sciences, Karaj, Iran *

**Keywords:** *Leishmania*, LPG3, Natural killer cell, TLR

## Abstract

***Background:*** Natural killer (NK) cells play an important role in early stages of innate immune responses against viral and tumoral attacks. Activation of NK cells by leishmaniasis results in secretion of cytokines such as interferon (IFN)-γ and tumor necrosis factor (TNF)-α, which enhance the phagocytosis and clearance of parasite. Lipophosphoglycan 3 (LPG3), the *Leishmania* homologous with GRP94 (glucose regulated protein 94), a member of HSP90 family, contributes to LPG assembly as the most abundant macromolecule on the surface of *Leishmania *promastigotes.

***Methods:*** We purified NK cells from healthy individuals (n=10) using magnetic-activated cell sorting (MACS) technology. Purified NK cells were co-incubated with different concentrations of recombinant LPG3 (rLPG3), and its N-terminal (NT) and C-terminal (CT) fragments. Finally, the production of IFN-γ and TNF-α by NK cells were measured by ELISA.

***Results:*** Recombinant LPG3 but not its fragments (CT and NT), could significantly enhance the production of TNF-α by NK cells (*P*<0.05). Moreover, rLPG3, CT, and NT fragments were markedly stimulated the secretion of IFN-γ by NK cells (*P*<0.001).

***Conclusion:*** The *Leishmania* LPG3 antigen could effectively activate NK cells, in vitro.* Leishmania* LPG3 participates in the innate immunity against leishmaniasis and thereby improves the effective parasite destruction. However, its efficiency should be tested in vivo.

## Introduction

Leishmaniasis is an infectious disease caused by intracellular parasites of the more than 20 genus of *Leishmania* and occurs in tropical and sub-tropical area of worlds. The infection is endemic in 98 countries, affecting 12 million individuals and threatening 350 million people worldwide annually (1). Life cycle of parasites consists of two separate stages, including intracellular *Leishmania *aflagellate amastigote and extracellular flagellate promastigote. The extracellular promastigote replicates in the midgut of insect vector and amastigote forms infects the macrophages of human and other mammals (2, 3).

The clinical manifestation of leishmaniasis ranges from self-healing cutaneous lesions to fatal visceral infections. Mucocutaneous, visceral (known as kala-azar) and cutaneous (the most common) are three major forms of leishmaniasis. Cutaneous leishmaniasis (CL) is caused by *L. major*, *L. aethiopica* and *L. tropica* in the Old world and by *L. amazonensis*, *L. guyanensis*, *L. mexicana*and *L. braziliensis* in the new world (4, 5). According WHO report, the prevalence of CL is approximately 1–1.5 million new cases annually and mainly occurs in seven countries including Afghanistan, Algeria, Peru, Syria, Brazil, Saudi Arabia and Iran (1, 6). Visceral leishmaniasis (VL) is caused by *L. chagasi* in the New World and* L. infantum* and *L. donovani* in the Old World and occurs in Nepal, India, Sudan, Bangladesh and Brazil. Mucocutaneous leishmaniasis (MCL) is mainly caused by *L. braziliensis* and *L. panamensis *in the New World and occurs in Brazil, Bolivia and Peru (1).

The flagellated promastigote of *Leishmania* is mainly covered with a thick glycoconjugate including lipophosphoglycan (LPG) and GP63 zinc metalloprotease (7). LPG is the most abundant and important surface molecule of *Leishmania *promastigotes and contains a type-2 glycophosphatidylinositol (GPI) membrane anchor. LPG contributes in pathogenesis and survival of parasite in host cells and consists of phosphosaccharide-repeat domain, anchored to the cell surface of the parasite via a GPI anchor. LPG plays a central role in infection cycle, release in the midgut of the insect vector, resistance to complement, interface with macrophages uptake, resistance to oxidative attack and successful establishment of parasites. *Leishmania *LPG3 (an endoplasmic reticulum chaperon) is responsible for the synthesis of the LPG and modulate the host's immune responses which belongs to HSP-90/GRP94 family (7-9). Current control measures relay on chemotherapy including miltefosine, pentavalent antimonial, amphotericin B and paromomycinare. These therapeutic protocols are usually expensive and cannot be used efficiently by poor countries. Moreover, these drugs are associated with severe toxicity, increasing parasite resistance, difficult route of administration and poor efficacy in endemic regions (10).

The innate immune responses against *Leishmania* include Natural killer (NK) cells, cytokines and phagocytes (11). Recognition of pathogens by antigen presenting cells (APCs) such as dendritic cells leads to production of interleukin (IL)-12 that induce activation of NK cells and the secretion of tumor necrosis factor (TNF)-α and interferon (IFN)-γ, which are crucial for the control of pathogens. NK cells are one of important components of innate immune system that contribute in early innate immunity. Activated NK cells are involved in immune response against pathogens including intracellular bacteria, viruses and parasites. Activation of NK cells by *Leishmania *leads to production of IFN-γ and TNF-α that stimulate mononuclear phagocyte to kill the parasite via the generation of reactive oxygen or nitrogen intermediates (12, 13).

In the present study, we investigated the ability of recombinant *Leishmania* LPG3 in stimulation of secretion of IFN-γ and TNF-α cytokines by NK cells.

## Material and Methods

***NK cells isolation by magnetic-activated cell sorting (MACS)***

The fresh heparinized peripheral blood was obtained from totally 10 healthy subjects with no history of leishmaniasis after receiving their informed consent. Donors were not from endemic area and verified by leishmanin test previously. Peripheral blood mononuclear cells (PBMCs) were isolated from heparinized blood on a Ficoll gradient (Sigma, Missouri, USA), as described previously (14). NK cells were isolated by negative selection using the MACS NK cell isolation kit II (Miltenyi Biotech, Bergisch-Gladbach, Germany), according to the manufacturer's instructions. Briefly, PBMCs were washed twice, resulted pellet was resuspended in 40 μL of MACS buffer per 107 total cells, and 10 μL of NK cell biotin-antibody cocktail was added. After incubation for 5 minutes in the refrigerator (2−8 °C), 30 μL MACS buffer and 20 μL NK cell microbead cocktail were added per 10^7^ total cells. Cells were incubated for an additional 10 minutes in the refrigerator and the volume was adjusted to a minimum of 500 μL of buffer. Finally, magnetic separation was done with the autoMACS separator.

***NK cells purity assessment***

**Fig.1:**
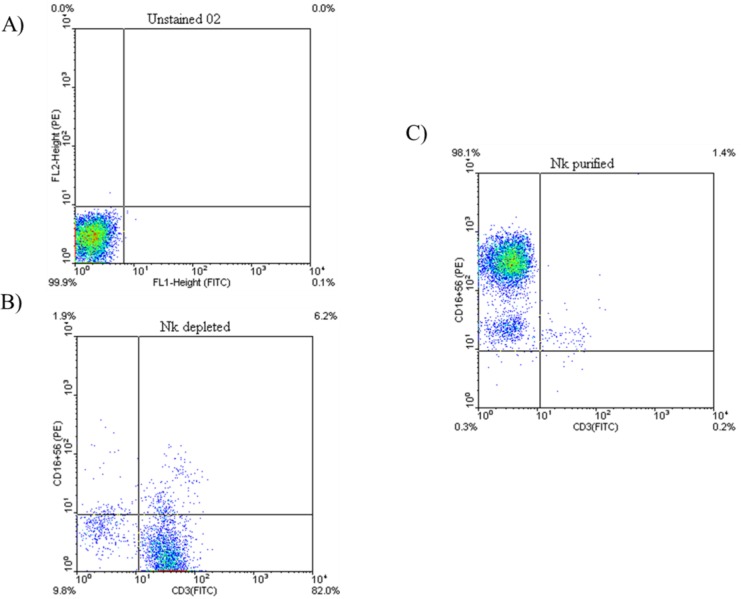
Representative dot plots demonstrating NK cells purity assessment. (A) Unstained cells used for evaluation of background staining. (B) The dot plots show NK depleted fraction which are highly CD3^+^. (C) The dot plots show purified NK cells which are CD3 ^-^CD16^+^CD56^+^

The purity of NK cells was checked by flow cytometry using fluorochrome-conjugated monoclonal antibodies (mAbs) anti-CD3 FITC and anti-CD16/CD56 PE (eBiosciences, San Diego, CA) and was usually greater than 90% (Fig. 1C). 

Briefly ,after twice washing with washing buffer (PBS 0.15 M, 0.5%BSA, 0.1% NaN3), 1 × 10^6 ^cells were resuspended in 100 μL washing buffer and stained with appropriate fluorochrome-conjugated mAbs and incubated for 40 min at 4◦C in the dark. Cells were then washed with wash buffer before scanning by flow cytometer (BD FACSCalibur). Data analysis was performed using the FlowJo software.

***Stimulation of Purified NK cells with different concentrations of LPG3, NT, and CT***

Recombinant LPG3 (rLPG3), N-terminal LPG3 (NT) and C-terminal LPG3 (CT) were produced at the Institute Pasteur of Iran as described previously (15) and were a generous gift from professor S. Rafati. 1×10^6^ NK cells were incubated with LPG3, NT and CT in three different concentrations (2, 10 and 20 µgr/ml) in 1 ml RPMI-1640 medium supplemented with 10% FBS for 48 h at 37 °C, 5% CO2.

***IFN-γ and TNF-α ELISA***

For analysis of the effects of LPG3, NT and CT on the production of IFN-γ and TNF-α by NK cells, cell-free culture supernatants were harvested and the concentrations of IFN-γ and TNF-α were determined by standard sandwich ELISA kits (R&D, Minneapolis, MN) according to manufacturer’s instructions. In brief, 96-well microtiter plates were coated with an unconjugated anti-TNF-α capture Ab or anti- IFN-γ capture Ab in 100mM Na2HPO4, pH 9.0 for 12 h at 4 °C, and blocked with PBS containing 0.05% Tween 20 and 10% FBS. Cell supernatants and recombinant human TNF-α standard or recombinant human IFN-γ standard (R&D Systems) were incubated in RPMI-1640 medium supplemented with 10% FBS for 2 h at room temperature. Bound TNF-α or IFN-γ were detected using a biotinylated mouse anti-human TNF-α Ab, or anti-human IFN-γ Ab in 1% BSA for 1 h. The plate was developed using streptavidin alkaline phosphatase conjugate with *P*-nitrophenyl phosphate (4 mg/ml) as substrate. The absorbance at 405 nm was read using a microtiter plate reader, and the concentrations of TNF-α and IFN-γ were calculated from a standard curve of recombinant human TNF-α and IFN-γ. The concentrations of both cytokines for each sample were calculated by regression analysis using the mean absorbance (average of triplicate readings of the sample added). For the cytokine production analysis, an intergroup comparison was performed by Kruskal-Wallis non-parametric ANOVA test. *P*-values below 0.05 were regarded as statistically significant.

## Results

***Recombinant LPG3, NT-LPG3 and CT-LPG3 induce IFN-γ production in NK cells***

**Fig. 2:**
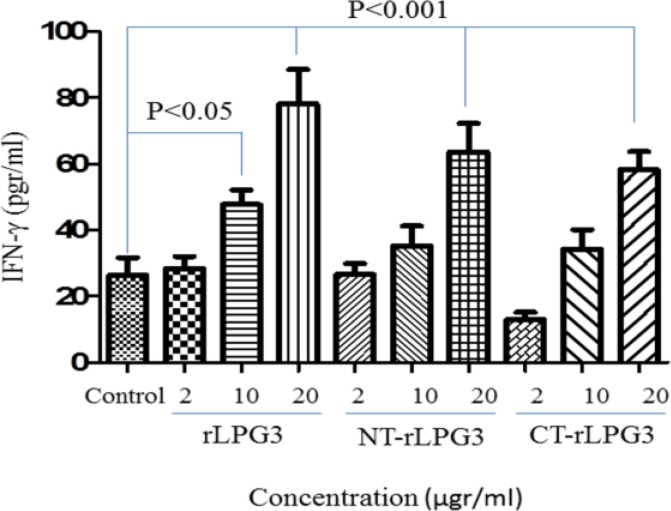
IFN-γ production by NK cells treated with different concentrations (2, 10 and 20μg/ml) of rLPG3 and its fragments (NT-LPG3 and CT-LPG3) after 48h incubation in comparison with untreated NK cells (control). LPG3 has the most stimulatory effect on IFN-γ secretion in both moderate (*P*<0.05) and high (*P*<0.001) concentration

The high concentrations (20 µg/ml) of LPG3 and its fragments could significantly increase the production of IFN-γ by NK cells (*P*<0.001). Moreover, the level of IFN-γ secretion was meaningfully higher LPG3 group with no differences for NT-LPG3 and CT-LPG3 groups in comparison with control (*P*<0.05). As shown in Fig. 2, rLPG3 has the highest stimulatory effect on IFN-γ secretion in both moderate and high concentrations when compared with NT-LPG3 and CT-LPG3 fragments.

***Recombinant LPG3 induces TNF-α production in NK cells***

**Fig. 3:**
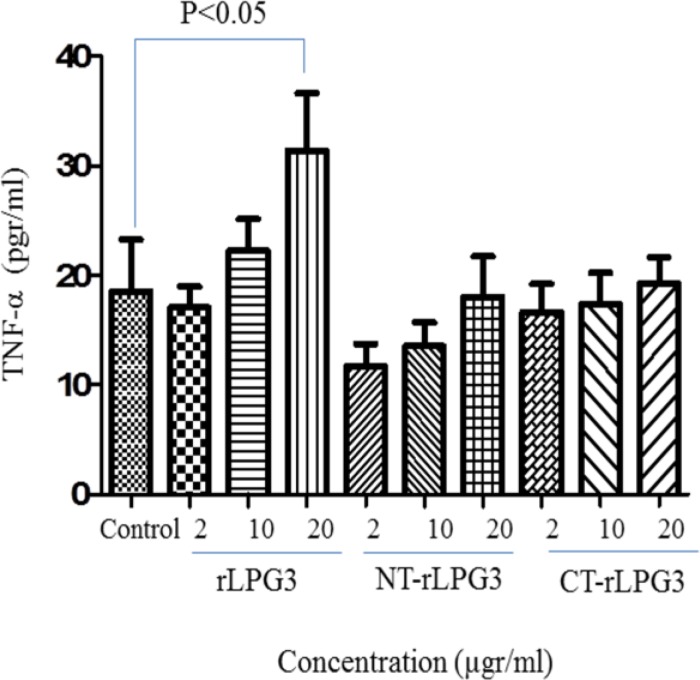
The levels of TNF-α secretions in co-cultured NK cells with various concentrations (2, 10 and 20μg/ml) of rLPG3, NT-LPG3 and CT-LPG3 compared with control. TNF-α increased significantly just following treatment NK cells with high concentration (*P*<0.05)

As shown in Fig. 3, while the recombinant LPG3 could significantly increase the production of TNF-α by NK cells, NT and CT fragments had no effects on secretion of TNF-α from NK cells even at the highest concentration (20 µgr/ml). Although the low and moderate concentrations of LPG3 (2 and 10 µgr/ml) could increase the TNF production by NK cells; however, the differences were not statistically significant (Fig. 3).

***Stimulatory effect of rLPG3 on NK cells is independent of TLR-2 signaling pathway***

**Fig. 4:**
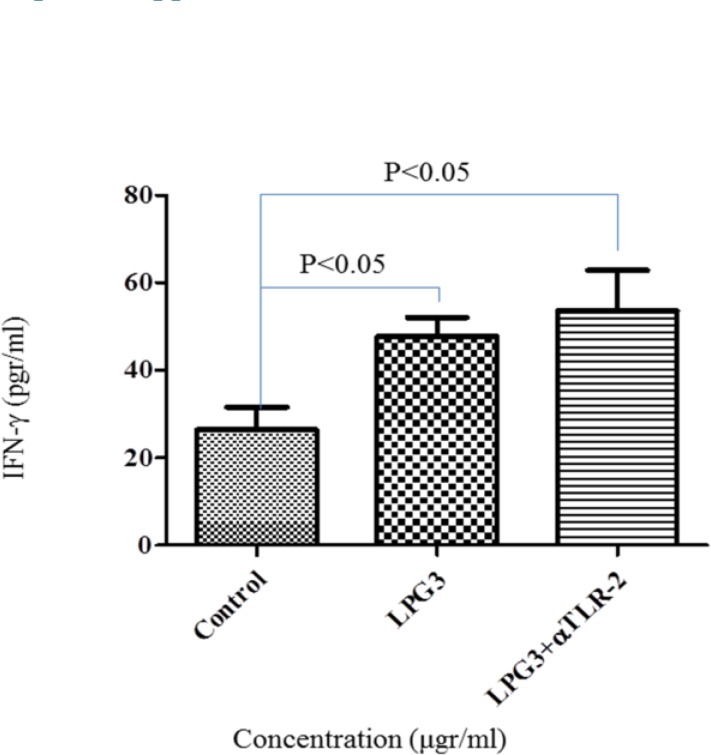
IFN-γ secretion in the presence or absence of anti- TLR-2 antibody. Human isolated NK cells were cultured with 10μg/ml of rLPG3 and its NT and CT fragments in the absence or presence of anti-TLR2 monoclonal antibody (αTLR-2) and the level of IFN-γ secretion was determined with ELISA

The effect of rLPG3 and its fragments is examined in the presence of Anti TLR-2 antibody to inhibit signaling pathway mediated by this receptor. Our results showed that, LPG3 and its NT or CT fragments have no difference induction effect on IFN-γ (Fig. 4) or TNF-α (data not shown) production when compared with the treatments in the absence of Anti TLR-2 antibody.

## Discussion

In the present study, we investigated the ability of recombinant *Leishmania* LPG3 in activation of NK cells. For this purpose, we isolated and purified NK cells from healthy individuals and co-incubated with different concentrations of LPG3, CT and NT fragments. Finally, the production of IFN-γ and TNF-α by NK cells were measured by ELISA. 

NK cells are an early source of IFN-γ that is crucial for innate immunity against *Leishmania* infection (13, 16-18). In lesions of patients suffering from diffuse cutaneous leishmaniasis, an untreated progressive form of the disease, NK cells are severely reduced and reemerged after treatment (19). Our result indicated that recombinant LPG3, CT and NT have a stimulatory effect on IFN-γ production by NK cells suggesting that IFN-γ production can be induced by both LPG3 and its fragments. Consistently, Rafati et al. (15) showed that the levels of IFN-γ in the supernatant of splenocytes obtained from mice immunized with LPG3-DNA were significantly higher in comparison with control groups (15). IFN-γ^−^^/^^−^ or IFN-γR^−^^/^^−^ C57BL/6 mice become highly susceptible to *L. major* infection, accompanied by an expansion of Th2-type responses (20, 21).

TLRs (Toll like receptors) as a pathogen recognition receptors (PRRs) expressed on the surface of T, B and NK cells which recognize pathogen associated molecular patterns (PAMPs) and have an essential role in the stimulation of innate immunity. Until now, 10 members of TLR family have been identified in human (TLR1_TLR10). Studies have revealed that TLRs recognize various ligands. For example TLR4 recognize LPS of Gram-negative bacteria, TLR2 recognize mycoplasma lipopeptide and lipoprotein and lipopeptide of Gram-positive bacteria (12, 13). 

In addition, NK cells elaborate TNF-α, which is contributed to elimination of intracellular pathogens (11, 22, 23). TNF-α has a potent function against mouse model of leishmaniasis (24). Other investigators showed that treatment with TNF-α led to reduction of lesion size and parasitic burden (25, 26). Furthermore, administration of anti TNF-α neutralizing antibody was associated with a transient aggravation of the disease (27, 28). TNF-α contributes to protective immunity by synergizing with IFN-γ to activate macrophages (29). IFN-γ and TNF-α stimulate macrophages to produce NO as an important effector molecule of innate and adaptive responses to *L. major* (30-32). We found that rLPG3 but not NT and CT fragments can stimulate NK cells to produce TNF-α. TNF-α production by NK cells only achieved at highest concentration of LPG3 (20 µgr/ml). On the other hand, LPG3 fragments (NT and CT) were not able to stimulate human NK cells to produce TNF-α indicating the necessity of whole LPG3 for TNF-α production.

Pirdel et al. showed a mixed Th1/Th2 response following immunization of BALB/c mice with the DNA encoding LPG3, which was associated with the secretion of both IFN- γ, and IL-10 by splenocytes in comparison with control groups (33). LPG3 and its fragment (rNT-LPG3) are highly immunogenic antigens and can induce both IgG1 and IgG2a production in BALB/c mice (15). 

Our results showed that recombinant LPG3 could induce NK cells to produce TNF-α. On the other hand, recombinant LPG3, NT-LPG3 and CT-LPG3 have a stimulatory effect on IFN-γ production by NK cell. Taking together LPG3 can stimulate immune system and inflammation through IFN-γ and TNF-α production through independent TLR2 signaling pathway. 

## Conclusion

Recombinant *Leishmania* LPG3 activates NK cells to secrete INF-γ and TNF-α cytokines that stimulate phagocytic cells to kill the parasite.
